# *Rickettsia prowazekii* and Real-time Polymerase Chain Reaction

**DOI:** 10.3201/eid1203.050888

**Published:** 2006-03

**Authors:** Sanela Svraka, Jean-Marc Rolain, Yassina Bechah, John Gatabazi, Didier Raoult

**Affiliations:** * Université de la Méditerranée, Marseilles, France;; † National Reference Laboratory, Kigali, Rwanda

**Keywords:** Rickettsia prowazekii, real-time PCR, epidemic typhus, bioterrorism, research

## Abstract

*Rickettsia prowazekii* is the causative agent of epidemic typhus and a potential bioterrorism agent. Sensitive and specific rapid assays are needed to complement existing methods of detecting this organism. We developed a real-time quantitative polymerase chain reaction assay by using a species-specific probe targeting the *gltA* gene. This assay, which was rapid, specific for *R. prowazekii* only, and sensitive (cutoff detection of 1 to 5 copies per sample), detected and directly identified *R. prowazekii* in blood of 12 experimentally infected mice sampled at day 3 and 6 postinfection or in naturally or experimentally infected lice. Because our assay is highly standardized and easily adaptable, it could improve epidemic typhus surveillance in public health programs, especially for countries with underdiagnosed or unrecognized human cases.

## Abstract 2

This highly standardized and adaptable assay could improve epidemic typhus surveillance.

*Rickettsia prowazekii* is the causative agent of epidemic or louseborne typhus, which is transmitted by the human body louse. This disease can be fatal and, without treatment with doxycycline, will cause death in up to 30% of cases (*1**–**3*). More than 30 million cases of epidemic typhus occurred during and immediately after World War I, resulting in an estimated 3 million deaths (*1*). Although the incidence of epidemic typhus is low today, the infection could reemerge in epidemic form in human populations, as recently reported in Burundi (*4*), Russia (*5*), and Algeria (*6*). Infections with *R. prowazekii* have been rarely reported in the United States: only 39 cases were reported from 1976 to 2001, all in persons who had no reported contact with body lice but did have contact with flying squirrels (*7**,**8*).

The ability to be acquired by the aerosol route, efficient arthropod transmission, and severe clinical outcome and death in untreated cases make *R. prowazekii* a category B bioterrorism agent. The former Soviet Union's Red Army developed *R. prowazekii* as a battlefield weapon, and the Japanese army successfully tested bombs containing the pathogen (*9*).

Since clinical signs of epidemic typhus are usually nonspecific, including fever, headaches, and severe myalgia, appropriate diagnostic methods are important (*10*). Despite recent developments in cell culture and molecular detection methods for the diagnosis of rickettsial diseases (*11*), serologic assays remain the simplest diagnostic tests to perform, even if serum samples are sent on filter paper (*12*). Nevertheless, serologic tests lack specificity because most also detect cross-reactive antibodies among the typhus-group rickettsioses. Moreover, a definite diagnosis of epidemic typhus is often delayed because the sensitivity of cell culture and polymerase chain reaction (PCR) methods is low (*13*), and serologic diagnosis can be obtained only by using advanced serologic methods such as Western blot analysis after cross-adsorptions. These methods are restricted to laboratories with biosafety level 3 (BSL-3) facilities and trained technicians (*14*). Recent studies have demonstrated the usefulness of PCR of body lice in ongoing surveillance of louse-associated infections, especially in outbreaks of epidemic typhus (*15*). The aim of our study was to develop a real-time quantitative PCR assay by using a species-specific probe that is rapid, sensitive, and specific for detecting *R. prowazekii* in clinical samples or in body lice in outbreaks of epidemic typhus.

## Materials and Methods

The *gltA* sequences of 22 *Rickettsia* species were aligned by using the multiple-sequence alignment program ClustalW supported by the Infobiogen website (http://www.infobiogen.fr). Within the alignments, primers and probe were selected that were specific for *R. prowazekii*.

*R. prowazekii* strain Breinl (ATCC VR-142) was grown in Vero cell monolayers cultured in minimal essential medium supplemented with 4% fetal calf serum and 2 mmol/L L-glutamine as previously described (*16*). Infected cells were harvested by using sterile glass beads and sonicated. Cell fragments were removed by centrifugation, and the supernatant was centrifuged for 10 min at 7,500 × *g*. The resulting pellet was resuspended in 20 mL phosphate-buffered saline, pH 7.5. *R. prowazekii* inoculum was quantified by using either the plaque assay method (*17*) or comparatively by 10-fold serial dilutions of a known plasmid standard of *R. prowazekii* containing 2.0 × 10^7^ copies per sample in an independent real-time PCR as previously described (*18*).

In this assay 4 *R. prowazekii* strains, 21 strains of *Rickettsia* spp., and 14 strains of bacteria from genera other than *Rickettsia* were evaluated (Table). We also included 31 lice from an outbreak of epidemic typhus in Rwanda in 2004 and 10 *R. prowazekii* laboratory-infected lice (*19*), 10 *R. typhi* laboratory-infected lice (*20*), and 10 *Borrelia recurrentis* laboratory-infected lice (*21*). Finally, we also included 30 lice received in June 2005 from Rwanda, which were tested only with the quantitative PCR (qPCR) assay (Table). Negative controls included 10 pathogen-free lice, distilled sterile water, and PCR mixture. All experiments were repeated 4 times. For mice samples, DNA samples extracted from blood of uninfected mice were used as negative controls.

**Table:** Strains used in real-time PCR*

Strains	Source	Standard PCR	LC PCR assay
*Rickettsia prowazekii* Breinl	ATCC	+	+
*R. prowazekii* Evir	UR	+	+
*R. prowazekii* BatnaRp22	UR	+	+
*R. prowazekii* Russian sample	UR	+	+
*R. typhi* Wilmington	ATCC	+	–
*R. massiliae* MtuI	ATCC	+	–
*R. montanensis*	ATCC	+	–
"*R. aeschlimanii*"	UR	+	–
*R. massiliae* strain Bar 29	UR	+	–
*R. helvetica* C6P9	ATCC	+	–
*R. felis*	UR	+	–
"*R. sibirica mongolitimonae*"	UR	+	–
*R. rickettsii*	ATCC	+	–
*R. conorii moroccan*	ATCC	+	–
*R. sibirica sibirica* 246	ATCC	+	–
*R. conorii* subsp. *israelensis* CDC1	G.A. Dasch	+	–
*R. africae* ESF–5	UR	+	–
*R. japonica* YM	ATCC	+	–
Thai tick typhus rickettsia	G.A. Dasch	+	–
*R. slovaca*	UR	+	–
*R. conorii* subsp. *caspia* A-167	UR	+	–
*R. australis* Phillips	G.A. Dasch	+	–
*R. honei* RB	GRIC	+	–
*Rickettsia* sp. AT1	UR	+	–
*R. bellii* 369L42-1	D.H. Walker	+	–
31 lice from Rwanda (2004)	J. Bosco	+ (10/31)†	+(17/31)†
30 lice from Rwanda (2005)	J. Gatabazi	ND	+(5/30)
*R. prowazekii* laboratory-infected lice	UR	+ (10/10)	+(10/10)
*R. typhi* laboratory-infected lice	UR	+ (10/10)	–
*R. prowazekii*–infected BALB/C mice	UR	+ (12/12)‡	+ (12/12)‡
*B. recurrentis* laboratory-infected lice	UR	– (0/10)	– (0/10)
*Borrelia recurrentis*	ATCC	–	–
*Escherichia coli*	CIP	–	–
*Proteus mirabilis*	CIP	–	–
*Staphylococcus aureus*	CIP	–	–
*Streptococcus salivarius*	CIP	–	–
*Orientia tsutsugamushi*	CIP	+	–
*Streptococcus pyogenes*	CIP	–	–
*Mycobacterium xenopi*	CIP	–	–
*Chlamydia trachomatis*	Human isolate	–	–
*Propionibacterium acnes*	UR	–	–
HGE agent	ATCC	–	–
*Bartonella quintana* Oklahoma	ATCC	–	–
*Tropheryma whipplei* Twist	UR	–	–
*M. tuberculosis*	CIP	–	–

*PCR, polymerase chain reaction; LC, LightCycler; ATCC, American Type Culture Collection, Rockville, MD, USA; GRIC, Gamaleya Research Institute Collection; G.A. Dasch, Naval Medical Research Institute, Bethesda, MD, USA; UR, Unité des Rickettsies, CNRS UPRES A, Marseille, France; D.H. Walker, University of Texas, Galveston; CIP, Collection Institute Pasteur, Paris, France; HGE, human granulocytic ehrlichiosis. †Number of positive lice/total number of tested lice. ‡PCR for each mouse was positive in blood at days 3 and 6 postinfection (for cycle thresholds see results in text).

We have also tested *R. prowazekii*–infected mice by using a currently available experimental model similar to the previous model described for *R. typhi* (*22*). We used 7-week-old female BALB/C mice (Charles River Laboratories, Arbresle, France) that were maintained in cages with sterile food and water. All experiments were performed in a BSL-3 laboratory. Twelve mice were injected with 1.8 × 10^5^ PFU/mL *R. prowazekii* strain Breinl (ATCC VR-142), and 6 mice were injected with uninfected cells. The solution containing bacteria was injected into the retroorbital venous plexus over a period of 30 s. We collected 200 μL of blood from each mouse at day 3 postinfection (PI) and at day 6 PI and stored it in EDTA at –20°C for PCR.

Total genomic DNA from bacterial strains was extracted with the Qiagen QIAamp Blood Kit (Qiagen, Hilden, Germany), and lice DNA and blood and biopsy samples from infected mice were extracted by using the Qiagen QIAamp Tissue Protocol (Qiagen). PCR was performed by using a LightCycler instrument (Roche Biochemicals, Mannheim, Germany).The PCR mixture included a final volume of 20 μL with 10 μL of the Probe Master kit (Qiagen), 0.5 μL (10 pmol/μL) of each primer, 2 μL (2 μmol/μL) probe, 5 μL distilled water, and 2 μL extracted DNA. The amplification conditions were as follows: an initial denaturation step at 95°C for 15 min, followed by 40 cycles of denaturation at 95°C, annealing and elongation at 60°C for 120 s, with fluorescence acquisition in single mode. Each sample was also tested with a standard PCR that was performed on a PCR instrument (Eppendorf, Mastercycler, Hamburg, Germany) using primers of the *gltA* gene (*23*).

## Results

The *R. prowazekii* inoculum used in this study was of 1.8 × 10^5^ PFU/mL using the plaque assay quantification method (*17*) and contained 1.16 × 10^6^ copies per sample when quantified with our plasmid standard (*18*). The selected primers and probe of the *gltA* gene specific only for *R. prowazekii* were as follows: RproF (5´-TCGGTAAAGATGTAATCGATATAAG-3´), RproR (5´-CATATCCTCGATACCATAATATGC-3´) and Rp.probe (FAM-ACTTTACTTATGATCCGGGTTTTATG-TAMRA), leading to a PCR product size of 154 bp. When qPCR assay was used, only *R. prowazekii* strains were positive, whereas the standard PCR assay detected all rickettsial species (Table). The standard PCR assay was positive for all 20 laboratory-infected lice (*R. typhi* or *R. prowazekii*), while qPCR assay was positive only for the 10 *R. prowazekii* laboratory-infected lice. Finally, blood samples obtained from our experimental model of *R. prowazekii*–infected mice at days 3 and 6 PI were also positive by using the protocol described above. The mean number of cycle thresholds (Ct value) for mice sampled at day 3 PI was 32.47 ± 2.11; at day 6 PI, the Ct value was of 35.52 ± 2.01 (p = 0.001). All uninfected lice, *B. recurrentis*–infected lice, and mice samples were negative with both assays.

The sensitivity of qPCR and the standard PCR was determined by using 10-fold serial dilutions of our known *R. prowazekii* inoculum (1.16 × 10^6^ DNA copies per sample). The sensitivity of the qPCR was increased 10-fold over that of the standard PCR. Compared to our plasmid standard, the cutoff detection of the qPCR was 1–5 copies per sample, whereas the cutoff detection was >10 copies for the standard PCR.

Among the 31 lice from Rwanda sampled in 2004, 17 were positive by real-time PCR, whereas only 10 of these 17 lice were positive by standard PCR. The latter 10 samples had a mean number of 1,300 DNA copies (Ct value 26.82–35.22). The 7 samples positive only by real-time PCR had a mean number of 8.5 DNA copies (Ct value 33.72–38.73). The real-time PCR therefore appears to be more sensitive. However, this difference was not significant (p = 0.07) perhaps because of the small number of tested lice. Finally, 5 of the 30 lice received from Rwanda in June 2005 were positive when the qPCR was used (Table).

## Discussion

We developed a real-time quantitative PCR for specific detection of *R. prowazekii*. The selected primers and probe were 100% complementary to *R. prowazekii* only and to no other rickettsial strains. We confirmed the specificity of these primers and probe on rickettsial isolates and other common bacteria and repeated the experiments 4 times without discrepancies. Real-time quantitative PCR for rickettsiae was first developed to test antimicrobial drug susceptibility (*24*) and then was used to detect *R. rickettsii* and closely related spotted fever group rickettsiae (*17*) or *R. prowazekii* strains (*25*).

Our assay has a greater sensitivity than the standard assay, with a cut-off detection of only 1 to 5 DNA copies per sample, as measured comparatively to plasmid DNA quantification. The sensitivity found with our standard PCR has been previously estimated at 1–10 DNA copies of the gene (*23*). The first use of standard PCR for detecting *R. prowazekii* using primers derived from the 17-kDa antigen sequence had a cut-off detection of as few as 30 rickettsiae (*26*). Cutoff detection of rickettsiae with real-time quantitative PCR ranges from 5 copies (*17*) to 10 copies (*25*). Using our LightCycler assay, we detected an extra 7 samples in lice from Rwanda as compared to standard PCR. Only 1 report exists of real-time detection of *R. prowazekii* using molecular beacon probes targeting the *ompB* gene (*25*). In this report, only 2 *R. prowazekii* strains were tested (*25*). Moreover, we showed that *R. prowazekii* can be amplified from blood of experimentally infected mice. This experimental model of *R. prowazekii* infection and the ability to quantify the bacteria with the real-time PCR could be used to better study the pathogenesis of the organism. We found in this mouse model that the number of bacteria in blood was lower at day 6 PI than that at day 3 PI, which suggests that mice can eradicate infection at this dose.

The assay we describe can be performed wherever a real-time quantitative PCR machine is available. The reagents and the machine are standardized; this method gives rapid results (sequencing is not necessary) and decreases the likelihood of error. This assay was applied successfully in lice received from Rwanda in June 2005. Indeed, using our assay we were able to alert the World Health Organization of the presence of *R. prowazekii*–positive lice within 1 working day.

Because body lice and their associated diseases are generally encountered in areas where medical and biologic assistance is limited, local assessment of their roles as sources of infection is difficult. Lice are easy to collect and to transport to reference laboratories, where suitable molecular biologic approaches can be used (*23*). Although sucking lice die within 24 h of their final blood meal, the infecting bacterial DNA will remain intact for extraction for several weeks if the samples are kept dry (*15*). Upon arrival in the laboratory, the lice can be processed very quickly, and a diagnosis can be established rapidly (DNA extraction and LightCycler PCR take ≈5 h). Several weeks are necessary to obtain bacterial culture and serologic results, and those procedures do not always highlight the presence of bacteria. The usefulness of bacterial DNA detection in lice by PCR has been demonstrated by recent investigations. In central Africa, large outbreaks of lice infections occurred during civil wars in Burundi, Rwanda, and Zaire and preceded the outbreak of epidemic typhus by 2 years (*4*). Finally, our data obtained in experimentally infected mice suggest that real-time PCR could also be useful for detecting *R. prowazekii* directly from blood specimens. Because our assay is highly standardized and easily adaptable anywhere and anytime, it could improve epidemic typhus surveillance in public health programs, especially for countries with underdiagnosed or unrecognized human cases (*4*).
